# Design and Analysis of Handshake-Based MAC with Delay Variations in Underwater Acoustic Networks

**DOI:** 10.3390/s19194159

**Published:** 2019-09-25

**Authors:** Chao Dong, Yankun Chen, Quansheng Guan, Fei Ji, Hua Yu, Fangjiong Chen

**Affiliations:** 1 South China Sea Marine Survey and Technology Center, Guangzhou 510300, China; dongchao@smst.gz.cn (C.D.); chenyankun@smst.gz.cn (Y.C.); 2 Key Laboratory of Technology for Safeguarding of Marine Rights and Interests and Application, Guangzhou 510300, China; 3 South China University of Technology, Guangzhou 510300, China; eefeiji@scut.edu.cn (F.J.); yuhua@scut.edu.cn (H.Y.); eefjchen@scut.edu.cn (F.C.)

**Keywords:** underwater acoustic networks, medium access control, delay variations

## Abstract

The long propagation delay in underwater acoustic channels has attracted tremendous attentions in designing Medium Access Control (MAC). The low acoustic propagation speed and wide area of the acoustic communication range led to a wide range of variations in the propagation delay. This paper identifies an important characteristic of two-scale delay variations by field test results. We carry out simulations to study the impact of delay variations on MAC, and the results suggest a slot length adaptation scheme for the handshake and slotting based MAC. We further model an absorbing Markov chain to derive the closed-form equation for the throughput of MAC with adaptive slot length. Both the analytical and simulation results show that our proposed slot length adaptation improves significantly the throughput of MAC in underwater acoustic networks. Particularly, the Slotted-FAMA with an adaptive slot length achieves more than double the throughput than the Slotted-FAMA with a fixed slot length in a network with six nodes.

## 1. Introduction

Underwater acoustic networks (UANs) are growing rapidly and receiving attention. They have been found in some military and commercial applications, like disaster prevention, tactical surveillance, offshore exploration, pollution monitoring, and oceanographic data collection [1,2].

Medium access control (MAC) enables channel sharing for multiple nodes in UANs. Different from the terrestrial radio frequency waves, the underwater acoustic waves propagate slowly at ~1500 m/s, leading to a long propagation delay that is five times longer than the radio propagation. The long propagation delay has be noticed in the literature. It was found that the long propagation degrades Slotted-ALOHA to pure ALOHA, which leads to throughput losses [3,4,5]. Many works have tried to improve ALOHA or CSMA to consider the long propagation delay [4,6,7,8,9].

**Figure 1:**
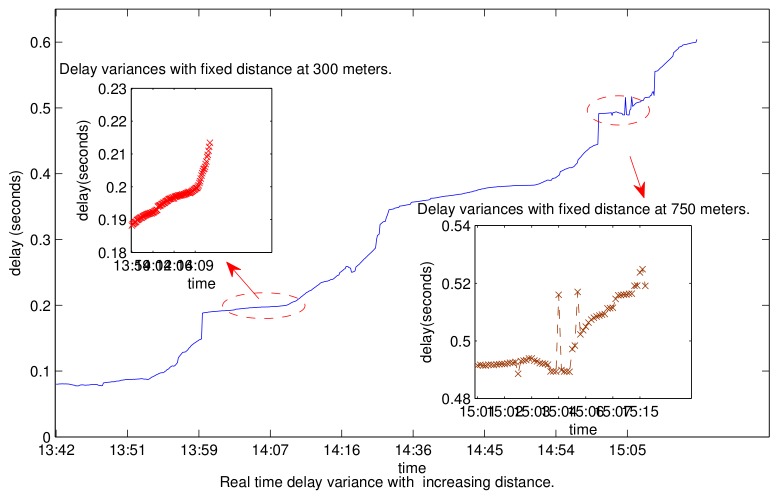
The acquisition of the two scale delay.

We note that the long propagation delay also varies within a wide range due to the low acoustic propagation speed and the dynamic ocean environment. There exist large-scale and small-scale delay variations in the underwater environment. The large-scale delay variation is generated by the different geographic distributions of nodes. For example, the delay varies from 1 second to 2 seconds for the propagation distances from 1.5 kilometers to 3 kilometers at the propagation speed of 1500 m/s. The small moves of transceivers, the drift with the wave, the variation of the propagation speed, and multipath propagation can cause a small variation of propagation delay, which is however not negligible. A small move of 100 m will cause a variation of 2 × 67 milliseconds.The multiplier 2 means both the sender and the receiver may move in the opposite direction from each other. Meanwhile, the delay increases by almost 69 ms when the propagation speed decreases from 1500 to 1450 m/s at a distance of 3 kilometers. The underwater acoustic speed has a range from 1450 m/s to 1540 m/s, depending on the salinity in ppt, the temperature in degree centigrade, and the depth [1]. We carried out our field test at Longdong Reservoir, Guangzhou. We equip each boat with an AquaSeNT modem. The transmitting power of AquaSeNT is −20 dB, the receiving gain is 0 dB, and the carrier frequencies are between 21 K and 27 K. We fix one boat, and move the other boat slowly to get the real-time boat-to-boat distance and observe the large-scale delay variations. We fix two boats to get more data to observe the small-scale delay variances at two fixed distances. Our field test results in this paper have verified the above multiscale delay variations, as shown in Figure 1.

Handshake-based and slotting based MAC protocols, e.g., Slotted Floor Acquisition Multiple Accesses (Slotted-FAMA) [10], are more sensitive to delay variations. Handshake-based protocols require accurate estimation of round-trip time (RTT) to carry our channel reservation [11]. Unnecessary channel reservation may be triggered due to the wrong estimation of RTT [12]. To avoid transmission collisions between adjacent slots, the slot length must be set to the largest propagation delay, which corresponds to the maximum distance between nodes in the network, though a large slot length may lead to a low channel utilization. The fixed slot length in Slotted-FAMA may also lead to the failures of the two-way handshakes due to the dynamic delay variations, which includes both the large-scale and small-scale delay variations.

This paper studies the impact of delay variations on Slotted-FAMA, which has exhibited high capability in avoiding collisions, via both simulation and modeling. The contributions of this paper are summarized as follows.

- Adaptive slot length for Slotted-FAMA: We first identify the multiscale delay variations using field tests. We then carry out simulations to show that the delay variations degrade the throughput of Slotted-FAMA with the fixed slot length, and the peak throughput can be achieved at a slot length of the actual propagation delay. Based on this observation, we propose an adaptive slot length to overcome the effect of the delay variances to improve the throughput of MAC protocols.
- Absorbing Markov chain modeling: We model the Slotted-FAMA with adaptive slot length by an absorbing Markov chain and derive the closed-form expression for the throughput.
- Analysis and simulation studies: The simulations using the Aqua-Sim [13] in ns2 validate our absorbing Markov chain modeling. Simulations are also carried out to verify the throughput improvement of our proposed adaptive slot length.

The rest of this paper is arranged as follows. Section 3 motivates our study of delay variations via simulations and proposes the slot length adaptation. Section 4 models the handshake and slotting based MAC by an absorbing Markov chain and derives the closed-form throughput. Section 5 verifies our analytical model by simulations. Finally, Section 6 concludes this paper.

## 2. Related Works

Generally, MAC protocols are mainly divided into two categories: contention-free protocols and contention-based protocols. Contention-free protocols are divided into time division multiple access (TDMA), frequency division multiple access (FDMA), and code division multiple access (CDMA), according to the channel divisions in time, frequency, and orthogonal codes. Due to the limited bandwidth of underwater acoustic channels and the vulnerability of limited-band systems to fading and multipath, the pure FDMA technique is not suitable for UANs [14,15]. TDMA has lower channel utilization and has difficulty in realizing precise synchronization due to the large propagation delay [15,16,17,18]. Although CDMA is a promising medium access technique allowing simultaneous transmissions via using pseudo-random codes, the CDMA-based MAC protocols face the inherent weakness on the near–far effect, which is the major open issue for underwater acoustic channels.

Contention-based MAC protocols are mainly classified into random access protocols and handshake-based protocols. There exists two typical random access methods, i.e., ALOHA [6,19,20] and Carrier Sensing Multiple Access (CSMA) [7,21]. ALOHA allows nodes to send packets whenever they have any, whereas CSMA needs to listen the channel before transmissions. Many ALOHA-like and CSMA-like protocols are designed for the terrestrial radio networks because the radio propagation delay is very small. Directly applying the terrestrial MAC protocols into UANs is not feasible since the underwater acoustic propagation delay is five-order longer than the radio propagation. Several variants of ALOHA are proposed to avoid the collisions due to long propagation delay [6]. ALOHA combined with CSMA is also proposed to avoid the collisions with ongoing transmissions in the work by the authors of [6]. Chirdchoo et al. developed several variants of ALOHA in [6] to avoid collisions due to long propagation delay. In the work by the authors of [6], carrier sensing was used to avoid the sensed ongoing transmissions in ALOHA-CS and ALOHA-HD. ALOHA-CA and ALOHA-AN try to establish the collision database by exchanging the location information among neighbors. A similar technique was also adopted in Propagation Delay Aware Protocol (PDAP) [7] to achieve distributed transmission scheduling.

The underwater acoustic MAC has merited analysis by researches. In the work by the authors of [22], the single-hop saturation throughput of slotted Bic-MAC in UANs based on Markov chain was presented. In addition to the long propagation delay, Zhu et al. also noticed that the delay introduced by the long preamble in the practical acoustic signal degrades the performance of existing underwater acoustic MAC protocols [5]. The authors analyzed the impact of the long preamble on acoustic MAC, and proposed a time-sharing-based MAC to achieve better performance. So far, the impact of delay variations on handshake-based MAC protocols for UANs has attracted less attentions in both simulation and modeling analysis. Thus, we should understand how the delay variations affect the performance of MAC before designing an efficient acoustic MAC.

The essential idea of handshake-based MAC protocols is to reserve the channel before sending data packets. The long propagation delay also has an important impact on handshakes of control packets in UANs. It was shown in Multiple Access Collision Avoidance for Underwater (MACA-U) [9] that the long propagation delay may improve the MAC performance. For example, a persistent “waiting for CTS strategy” can enable concurrent transmissions. Deferring data transmissions after receiving the CTS can also avoid potential interference in Distance-Aware Collision Avoidance Protocol (DACAP) [21], which uses distance information to determine the deferring time. The authors of [23,24] found that the collisions in UANs depend on the transmitter time and receiver location, which is called space-time uncertainty. Although this uncertainty reduces the effectiveness in detecting the ongoing transmissions, it enables contender detection and counting, and thus improves channel reservation in Tone-Lohi (T-Lohi). Due to the asynchronous arrivals of RTS/CTS frames on different nodes at different times, Adaptive Propagation Delay Tolerant Collision Avoidance Protocol (APCAP) [8] delays the data transmissions to guarantee informing the potential interferers by RTS/CTS. During the waiting period, other frames are allowed to transmit to further improve the channel utilization. Bic-MAC [25] designs an asynchronous handshaking to conduct concurrent and bidirectional data exchanges to improve the channel utilization. Instead of RTS/CTS handshakes, UWAN-MAC [26] uses SYNC messages to announce the transmission cycles, and thus achieves distributed transmission scheduling. Frame slotting is another technique to avoid collisions. Slotted ALOHA [20] almost doubles the throughput in the terrestrial wireless networks by allowing transmissions only at the beginning of a time slot. Slotted-FAMA [10] adopts slotting technique to improve the handshake-based FAMA protocol for UANs.

However, the delay variation and its impact on the underwater acoustic MAC are seldom noticed in the literature. The authors of [12] pointed out that the delay variation caused by time-varying multipath propagation may prevent accurate estimation of the RTT. Zhang et al. [27] mentioned the delay variation in the simulation environment and scenario setting, but the study does not include the impact of the delay variations on MAC protocols. In [3], Joon et al. illustrated the impact of varying propagation latency on medium access, with ALOHA and slotted ALOHA protocols as a case study. The work by the authors of [3,4] also found that the throughput of Slotted-ALOHA degrades to pure ALOHA with delay variations. To overcome the throughput degradation, Mandal et al. [4] proposed increasing the slot length to accommodate the synchronization error in a modified receiver synchronized Slotted-ALOHA. The simulation result in the work by the authors of [4] showed that optimally increasing the slot length can improve the throughput, which is degraded by the propagation delay uncertainty.

## 3. Delay Variations Definition and Slot Length Adaptation

In this section, we motivate our study on Slotted-FAMA by simulations, and then propose an adaptive slot length to improve the throughput of Slotted-FAMA.

### 3.1. Overview of Slotted-FAMA

Slotted-FAMA exploits carrier sensing, RTS/CTS handshakes, and time slotting techniques to coordinate the sharing of underwater acoustic channel.

**Figure 2:**
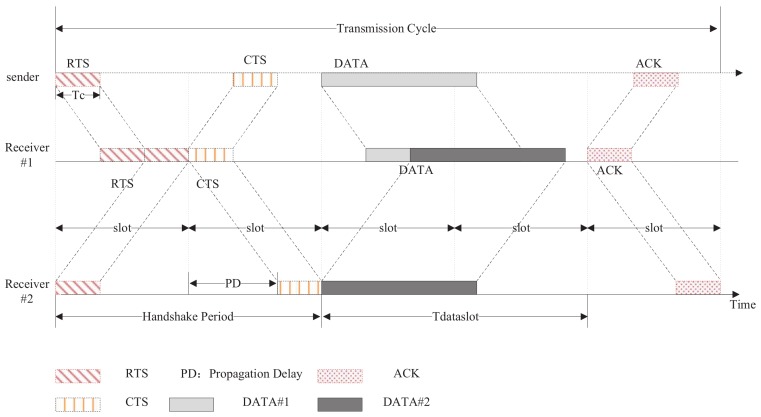
A transmission cycle in Slotted-FAMA.

As shown in the Figure 2, when the source node has to send a data packet, it waits until the beginning of the next slot and sends an RTS after carrier sensing. The destination node then sends back a CTS at the beginning of the next slot. When the source node has received the CTS, it will start sending the data packet at the beginning of the next slot. On receiving the data packet correctly, the destination node sends an acknowledgement (ACK) to indicate the successful transmission. Other terminals who overhear any RTS/CTS will trigger virtual carrier sensing (VCS), which defers their accessing the channel until the end of a duration indicated by RTS/CTS.

The slot length in Slotted-FAMA is set to the sum of the RTS/CTS transmission time and the maximum propagation delay, i.e.,

(1) $$T_{slot}=T_{c}+d_{max},$$

where $T_{c}$ is the transmission time of a control frame, e.g., RTS, CTS, or ACK, and $d_{max}$ equals to the result of the maximum propagation distance $D_{max}$ divided by the acoustic velocity *v*, i.e., $d_{max}=\frac{D_{max}}{v}$. The underwater acoustic velocity is often assumed as 1500 m/s in Slotted-FAMA.

### 3.2. Definition of Delay Variations

We use a simple network scenario with three nodes to illustrate the impact of delay variations. A receiver, *r*, is deployed at the location (0,0,0). The first transmitter, s1, is located at (1500 m,0,0), and the second transmitter, s2, is located at (3000 m,0,0). Thus, the propagation distances for s1 and s2 are 1500 m and 3000 m, respectively.

In addition to the low propagation speed, the propagation delay in the underwater acoustic channel varies due to two reasons:

- Large-scale delay variation: By assuming a fixed propagation speed at 1500 m/s, s1 needs 1 s to propagate its carriers to *r*, whereas s2 needs 2 s. In the practical applications, the medium transmitter–receiver distance has reached up to tens of kilometers [28]. In this sense, the propagation delay may range from hundreds of milliseconds to tens of seconds, which is caused by different geographic distributions.
- Small-scale delay variations: The locations of underwater nodes are not fixed. For example, the nodes may drift with the wave, and some vehicle nodes may cruise. Even a small move of 100 m has led to a delay variation of ~67 ms at the propagation speed of 1500 m/s. Furthermore, the salinity, temperature, and depth can change the acoustic speed in a range of [1450, 1540] [1]. For example, when the propagation speed decreases from 1500 m/s to 1450 m/s, s2’s carriers need 69 more milliseconds to reach its receiver. It is caused by a dynamic underwater environment.

To understand the impact of delay variations on Slotted-FAMA, we carry out simulation study over the above simple network scenario. We also add some random disturbance in the range of [−300,300] ms to each frame transmission to simulate the small-scale delay variation [27]. The slot lengths for s1 and s2 are the same. The data packet size is set to 3000 bits. The data rate is 1000 bits per second. The packet arrivals follow Poisson process, having an average arrival rate of 1 packet/s.

The throughput of MAC is obtained in the simulations as follows,

(2) $$\mathrm{Throughput}=\frac{\#\;\text{of received packets}\times T_{d}}{\mathrm{simulation}\;\mathrm{duration}},$$

where $T_{d}$ is the transmission time for a data packet.

**Figure 3:**
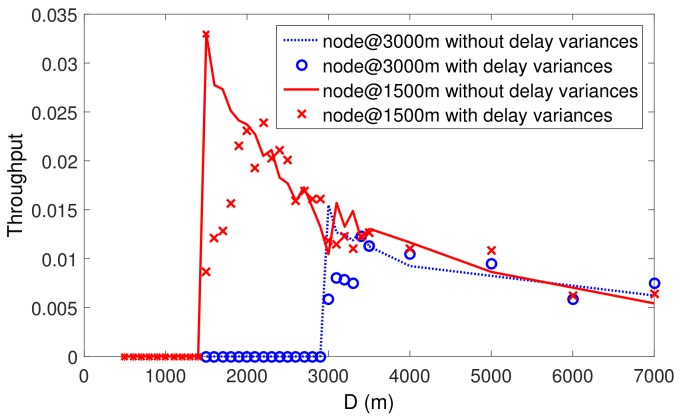
Impact of the large-scale and the small-scale delay variations on throughput of Slotted-FAMA. The throughput is obtained according to Equation (2).

Instead of setting the slot length with the maximum propagation delay (it equals 2 seconds in the simulated scenario for Figure 3). in the network, we take the $d_{max}$ as a variable and change $d_{max}$ to study the impact of delay variations on Slotted-FAMA. We have three observations from the simulation results in Figure 3:

- The throughput of s1 is almost zero when $d_{max}<1$ s, whereas the throughput of s2 is almost zero when $d_{max}<2$ s. The reason for this phenomenon is that Slotted-FAMA fails to reserve channel for data transmissions using RTS/CTS handshakes (Slotted-FAMA exchanges RTS/CTS before data transmissions to reserve channel). The RTS or CTS requires around 1 s to to be propagated between s1 and *r*. When $d_{max}<1$ s, the previous RTS/CTS transmissions may collide with RTS/CTS transmissions in the next slot. In this case, node s1 fails to reserve the channel to send the data. A similar phenomenon is also observed at node s2. This observation shows the impact of large-scale delay variation.
- Without small-scale delay variations in the simulation setting, nodes s1 and s2 reach their maximum throughput at around $d_{max}=1$ s and $d_{max}=2$ s, respectively. However, with small-scale delay variations, the throughput for both s1 and s2 reach their maximum values at around $d_{max}=1.33$ s and $d_{max}=2.33$ s instead. It takes an ~330 ms longer slot length to reach the peak of throughput with the small-scale delay variation in the range of [−300,300] ms. This is because the underwater acoustic communication needs a long preamble and a long processing time [5]. This observation shows the impact of small-scale delay variation.
- The throughput increases and then decreases with the increase of $d_{max}$. The maximum throughput is achieved when the slot length is set to around the actual propagation and transmission delay. The reason is that a short slot length (i.e., $d_{max}<1$ s for s1 and $d_{max}<2$ s for s2) may result in the channel reservation failures, whereas a long slot length wastes time in idle waiting and results in a low channel utilization. This observation shows the importance of adapting the slot length to the propagation delay variations.

The simulation results show the non-negligible impact of the large-scale and small-scale delay variations on the throughput of Slotted-FAMA, which motivates our study and suggests an adaptive slot length setting for Slotted-FAMA.

### 3.3. Adaptive Slot Length for Slotted-FAMA

The simulation results in the previous subsection have suggested an adaptive slot setting for Slotted-FAMA, i.e.,

(3) $$T_{slot}=T_{c}+d+\Delta t,$$

where $d=\frac{D}{v}$, *D* is the transceiver distance, and Δt is used to cover the frame processing time and the small-scale delay variation.

The slot length is determined in the way that ensures absence of packet collisions. The slot length should be at least the sum of the actual propagation delay, the transmission time of RTS/CTS/ACK packets, and the processing time. The large-scale propagation variation can be easily estimated by the transmitter–receiver distance and by assuming a propagation speed at 1500 m/s. Some extra guard should be also added to the slot length to cover the small-scale delay variation and the frame processing time. Instead of setting a fixed slot length as Equation (1), the slot settings in Equation (3) are different for different nodes. The setting of $d_{max}$ in Equation (1) is fixed for all the links, whereas the settings of *d* and Δt in Equation (3) vary for different links due to the delay variability. Thus, the slot length in Equation (3) is adaptive to the actual internodal propagation delay.

The adaptive slot setting in Equation (3) requires knowledge of *d* and Δt. The default of *d* can be known from the initial deployment of the network. The value of *d* can be updated by the internodal distance, which is estimated by localization technique in underwater sensor networks [29,30].

## 4. Throughput Modeling

This section establishes an absorbing Markov chain model for Slotted-FAMA with adaptive slot length, and derives the closed-form expression for the throughput. Markov chain has been a useful tool in analyzing MAC protocols, as in the work by the authors of [31], which established a Markov chain to analyze the throughput of IEEE 802.11 DCF. This section extends the Markov chain model to accommodate the propagation delay variations.

### 4.1. System Model

There are totally *N* nodes randomly located around the receiving node in the network. The packet arrival process is modeled as a Poisson process with an arrival rate of λ packets per second. The transmission time for each data frame is $T_{d}$.

The probability that a data packet is detected and decoded correctly is denoted by $K_{d}$. It depends on the detection probability of each packet (relating to the feature of the detecting preamble added at the start of each frame) and the decoding probability (relating to the bit error rate (BER) in the physical layer (PHY), packet length, and the coding scheme). Similarly, we denote the detection and decoding probability of a control packet by $K_{c}$, due to the different modulation schemes and packet lengths for control packets. The symbols used in the discussion are summarized in Table 1.

### 4.2. Markov Chain Model for Slotted-FAMA

To obtain a closed-form expression for the throughput in Equation (2), we need only consider a transmission cycle, as shown in Figure 2. Each transmission cycle ends with the data packet reception. Thus, we use an absorbing Markov chain to describe a transmission cycle.

#### 4.2.1. System States

Packet transmissions begin with State 1, and the terminal will carry out channel reservation after back-off. When a terminal detects the carrier on the channel, it will enter States 2–6 according to the different types of the overheard packets. Then, the terminal will carry out virtual carrier sense (VCS).

**Figure 4:**
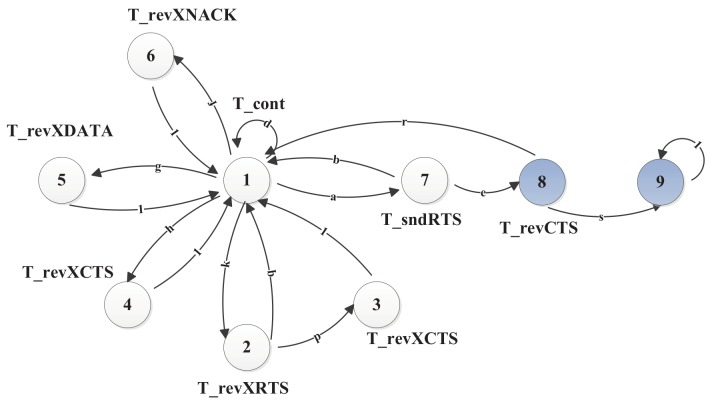
Absorbing Markov model for Slotted-FAMA.

When a packet arrives, the terminal goes to State 7, so as to initiate an RTS at the beginning of a slot, and waits for two consecutive slots to receive the response. The receiver will send back the CTS response at the start of the next slot if the RTS is successfully received. When CTS is successfully fed back, the terminal in State 8 will start sending the data packet in the next slot. Otherwise, the transmitter goes to the contention state. The terminal in State 9 receives the ACK, finishing the packet transmission. The absorbing Markov chain model and its state transitions are illustrated in Figure 4.

The absorbing Markov chain model accounts for the cycle of the RTS contention until the data packet is transmitted successfully. The absorbing State 9, as shown in Figure 4, makes it convenient to compute the expectation of the total duration of sending a data packet. Actually, the terminal will go to State 1 after successfully transmitting a data packet, and then start a new cycle for the next packet. All the states are summarized in Table 2.

The terminal may begin with any possible receiving states depending on the overheard packets before its data packet arrives from the upper layer. Otherwise, it will go the contention state (i.e., State 1) directly. Next, we calculate the state transition probability and state stay time.

#### 4.2.2. State Transition Probability

We now calculate the state transition probabilities for the absorbing Markov chain model. The state transition is represented by (m,n) for a transition from state *m* to *n*. State transition probability is represented by P(m,n). Table 3 lists all of the state transition probabilities. The state stay time for each state is marked under each state in Figure 4.

We have assumed Poisson packet arrivals with a rate λ and that Slotted-FAMA adopts a back-off with a constant contention window size *W*. It has been shown in the work by the authors of [32] that, given a constant back-off window *W*, a node has a probability of $\frac{2}{W+1}$ to access the channel. In another word, the node has a probability of $\frac{2}{W+1}$ to send an RTS. We then have the following probability of sending an RTS at the start of a new slot,

(4) $$P\left(1,7\right)=a=\left(1-e^{-\lambda T_{slot}}\right)\cdot \frac{2}{W+1}.$$

The transition from State 7 to State 8 happens in the condition that both of the RTS and CTS transmissions are successful in two consecutive slots. The ongoing RTS and CTS transmissions should not collide with any other transmissions. In this sense, the probability for a successful transmission is ${\left(1-a\right)}^{N-1}$, namely, no other terminal transmits in the same slot. Considering the receptions of both the RTS and the CTS with the same detection and decoding probability $K_{c}$, we have

(5) $$P\left(7,8\right)=e={\left(1-a\right)}^{\left(N-1\right)}K_{c}^{2}.$$

If the CTS is not successfully received, the terminal goes back to State 1. The transition probability is

(6) P(7,1)=b=1−e.

We have derived that a terminal has a probability of P(1,7)=a to send an RTS, thus the probability that a terminal does not send any RTS is 1−a. The terminal in State 1 will go to State 2 if it overhears an xRTS. The probability that one of the remaining N−1 neighbors has a successful RTS transmission is $C_{N-1}^{1}a{\left(1-a\right)}^{N-2}$. Further, with the reception probability $K_{c}$, the RTS is received successfully with a probability of P(1,2), which is calculated by

(7) $$P\left(1,2\right)=k=\left(1-a\right)C_{N-1}^{1}a{\left(1-a\right)}^{N-2}K_{c}=uK_{c},$$

where $u=\left(1-a\right)C_{N-1}^{1}a{\left(1-a\right)}^{N-2}$ is the probability of sending an RTS packet without collisions.

On receiving an xCTS, it will go to State 3 to carry out a VCS of $T_{revXCTS}$. After the VCS, it will then go back to State 1 with probability 1 to continue the contention for channel. We get P(2,3) as

(8) $$P\left(2,3\right)=p=K_{c}^{2}.$$

Correspondingly, we get P(2,1)=q=1−p.

State 4 overhears an xCTS, but misses the xRTS with a probability $1-K_{c}$. In this case, the RTS transmission for the intended receiver is successfully with a probability in Equation (7). Thus, we have the transition probability as

(9) $$P\left(1,4\right)=h=u\left(1-K_{c}\right)K_{c}K_{c}.$$

The terminal in State 4 will enter VCS for $T_{revXCTS}$ and return to State 1 with a probability of 1 after timeout.

The terminal transfers to State 5 from State 1, when the terminal overhears an xDATA packet. In this case, this terminal misses both the xRTS and xCTS. The overheard data packet also indicates the correct reception of both RTS and CTS at the intended receiver. Then we have P(1,5) as

(10) $$P\left(1,5\right)=g=uK_{c}^{2}{\left(1-K_{c}\right)}^{2}K_{d}.$$

The overheard NACK in State 1 triggers the transition to State 6, which means the successful transmissions of RTS and CTS, as well as the failure of the data packet. Then the transition probability is

(11) $$P\left(1,6\right)=f=uK_{c}^{2}\left(1-K_{d}\right){\left(1-K_{c}\right)}^{2}\left(1-K_{d}\right)K_{c}.$$

The terminal remains in State 1 if neither control packets nor data packets are received and no data packet arrives. The transition probability is

(12) P(1,1)=d=1−k−h−g−f−a.

The terminal will step from State 8 to State 9 when successfully receiving ACK with the following probability

(13) $$P\left(8,9\right)=s=K_{d}K_{c}.$$

#### 4.2.3. State Stay Time

Now we analyze the state stay time.

Any packets can only be sent at the beginning of a slot. Thus, the stay time for State 1 is

(14) $$T_{1}=T_{cont}=T_{slot}.$$

The terminal re-sends the RTS in State 7, if the corresponding CTS is not received. No matter whether the feedback CTS is received, the terminal will stay at least two slots in State 7. Then we have

(15) $$T_{7}=T_{sndRTS}=2T_{slot},$$

On receiving the CTS, the data transmission begins. Let $T_{dataslot}$ be the slot time needed by the transmission of a data packet, which includes the transmission time and the propagation time, i.e., $T_{dataslot}=\left\lceil \left(T_{d}+\frac{D}{v}\right)/T_{slot}\right\rceil \times T_{slot}$. The stay time of State 8 includes the transmissions of the data packet and ACK, that is,

(16) $$T_{8}=T_{revCTS}=T_{slot}+T_{dataslot}.$$

States 3, 4, and 6 share similar cases with State 8 in awaiting data packet transmissions. Then, these states have the same stay time, i.e., $T_{3}=T_{4}=T_{revXCTS}=T_{6}=T_{revXNACK}=T_{slot}+T_{dataslot}$.

In State 2, the terminal waits for CTS for

(17) $$T_{2}=T_{revXRTS}=2T_{slot}.$$

For the $T_{revXDATA}$ in State 5, the duration should be long enough to allow the reception of subsequent ACK or NACK packet, as there exists the possibility that the terminal can not hear the acknowledgment packet. It will wait an additional slot to detect whether the data packet is resent or not. Thus, the stay time for State 5 is

(18) $$T_{5}=T_{revXDATA}=T_{dataslot}+2T_{slot}.$$

The Markov chain model in Figure 4 is represented by the following state transition matrix,

(19) $$P\;=\;\left[\;\begin{array}{ccccccccc} d & k & 0 & h & g & f & a & 0 & 0\\ q & 0 & p & 0 & 0 & 0 & 0 & 0 & 0\\ 1 & 0 & 0 & 0 & 0 & 0 & 0 & 0 & 0\\ 1 & 0 & 0 & 0 & 0 & 0 & 0 & 0 & 0\\ 1 & 0 & 0 & 0 & 0 & 0 & 0 & 0 & 0\\ 1 & 0 & 0 & 0 & 0 & 0 & 0 & 0 & 0\\ b & 0 & 0 & 0 & 0 & 0 & 0 & e & 0\\ r & 0 & 0 & 0 & 0 & 0 & 0 & 0 & s\\ 0 & 0 & 0 & 0 & 0 & 0 & 0 & 0 & 1 \end{array}\right].$$

We can observe from the transition matrix that the process of sending a packet in Figure 4 is an absorbing Markov chain with State 9 as the absorbing state and other states as the transient states. We can get the state transition probabilities in **Q** for the transient states as

(20) $$Q\;=\;\left[\;\begin{array}{cccccccc} d & k & 0 & h & g & f & a & 0\\ q & 0 & p & 0 & 0 & 0 & 0 & 0\\ 1 & 0 & 0 & 0 & 0 & 0 & 0 & 0\\ 1 & 0 & 0 & 0 & 0 & 0 & 0 & 0\\ 1 & 0 & 0 & 0 & 0 & 0 & 0 & 0\\ 1 & 0 & 0 & 0 & 0 & 0 & 0 & 0\\ b & 0 & 0 & 0 & 0 & 0 & 0 & e\\ r & 0 & 0 & 0 & 0 & 0 & 0 & 0 \end{array}\right].$$

With the transient state matrix, we have the fundamental matrix as [33]

(21) $$N={\left(I-Q\right)}^{-1},$$

where the entry $n_{ij}$ of **N** gives the expected number of times that the transient state *j* is visited if the transition starts from the transient state *i* before being absorbed. Here, **I** is an identity matrix.

The transient probability of visiting the transient state *j* when starting from a transient state *i* is the (i,j)-entry of the following matrix **H**,

(22) $$H=\left(N-I\right)N_{dg}^{-1},$$

where $N_{dg}^{-1}$ is the diagonal matrix with the same diagonal as **N**.

### 4.3. Throughput Calculation

The throughput, which is denoted by *S*, can be defined as

(23) $$S=\frac{\bar{U}}{\bar{B}+\bar{I}},$$

where $\bar{B}$ is the expected duration of the busy period and $\bar{I}$ is the expected duration of the idle period. The sum $\bar{B}$ + $\bar{I}$ is the expected time of a transmission cycle. $\bar{U}$ is the expected time occupied by sending the useful data during a transmission cycle.

The terminal is busy in States 2 to 8. Particularly, States 2 to 6 are occupied by the ongoing transmission of other terminals, while the terminal sends its own data in States 7 and 8. The time between two consecutive data packet arrivals is considered as the idle time in State 1. Thus, we have

(24) $${\bar{T}}_{total}=\bar{B}+\bar{I}=\sum_{i=1}^{8}T_{i}H\left(1,i\right)N\left(1,i\right).$$

The successful transmission that makes a transition from State 8 to State 9 accounts for the value of $\bar{U}$, which is obtained by

(25) $$\bar{U}=e\cdot T_{d}\cdot K_{d}.$$

Finally, we get the throughput as

(26) $$S=\frac{e\cdot T_{d}\cdot K_{d}}{\sum_{i=1}^{8}T_{i}H\left(1,i\right)N\left(1,i\right)}.$$

## 5. Simulations and Discussions

In this section, we first use the original Slotted-FAMA to study the impact of delay variations and verify our analysis model, using Aqua-Sim [13] in *ns2*. Then, we compare the performance of the Slotted-FAMA with and without adaptive slot length under delay variations.

In the simulations, the data packet size is set to 3000 bits. The data rate is 1000 bits per second. We use Equation (2), which has the same physical meaning to Equation (23), to obtain the throughput in the simulations.

### 5.1. Impact of Small-Scale Delay Variations on Throughput

In the simulation, the receiver node is located at the origin (0,0,0). To focus on studying the small-scale delay variation, seven transmitter nodes are randomly deployed around the receiver node within a radius of only 500 m. The implementation of analysis is based on Equation (26). The slot length in Slotted-FAMA is computed as Equation (1), and the adaptive slot length for Slotted-FAMA is computed as Equation (3), where *d* is the actual distance between nodes. Thus, the largest internodal distance in the network is 500 m, and $d_{max}$ in Equation (1) for Slotted-FAMA should be set to 0.333 s. The small-scale delay variation is selected uniformly from −300 ms to 300 ms. This is because a small drift of 100 m has led to a delay variance of ~67 ms at the speed of 1500 ms, due to the low underwater acoustic propagation speed. In addition to the drift of nodes, the change in the propagation speed also causes propagation delay. The underwater acoustic propagation speed is changing in a range of [1450, 1540] due to the differences in salinity, temperature, depth, etc. There may be a 200 ms delay variance for 5 km from 1450 m/s to 1540 m/s.

**Figure 5:**
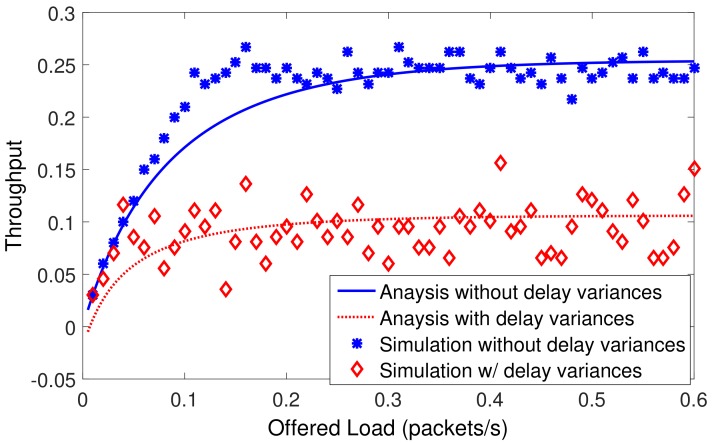
Impact of small-scale delay variation on throughput. The small-scale delay variation decreases the throughput of Slotted-FAMA.

The throughput of Slotted-FAMA with and without delay variations are both plotted in Figure 5. As shown in Figure 5, the throughput increases along with the offered loads, and reaches its saturated maximum at a high load of 0.3 packets/s. According to Equation (26), this is attributed to the decreasing idle time as the packet load gets high. At the saturated load, the idle time becomes small and the busy time dominates the idle time in determining the throughput. Figure 5 also shows that the delay variation decreases the throughput of MAC. This result is the same as the argument in [12]. The gap between the curves with and without the delay variations gets greater when the offered load increases, which means that the impact of delay variation becomes more significant at the saturated load. The simulation and analytical results match with each other in Figure 5, which also validates the correction of our analytical model.

### 5.2. Impact of Large-Scale Delay Variations on Throughput

**Figure 6:**
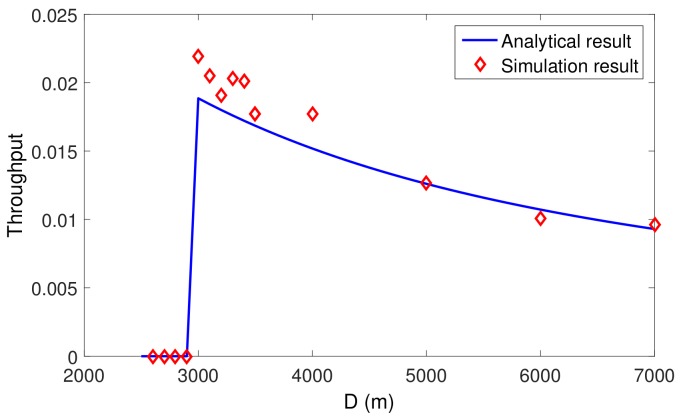
Impact of large-scale delay variation on throughput. The slot length in Slotted-FAMA should be slightly larger than the delay variation to reach the peak throughput.

Similarly, the implementation of the analysis is based on Equation (26), where the slot length in Slotted-FAMA is computed as Equation (1) and the adaptive slot length for Slotted-FAMA is computed as Equation (3), where *d* is the actual distance between nodes. We set two nodes with a distance of 3000 m to study the impact of the large-scale delay variation. We vary $d_{max}$ in Equation (3) for Slotted-FAMA from 1.667 s to 4.667 s. The small-scale delay variation is set to zero in the simulation setting. From Figure 6, we can see that the receiver can not receive any packets when d<2 s, because the transmitter fails to reserve the channel within two consecutive slots and hence has no chance to send the data. The throughput reaches the peak value at around d=2 s and then decreases when d>2 s, because of the idle-waiting time when using a long slot length.

**Figure 7:**
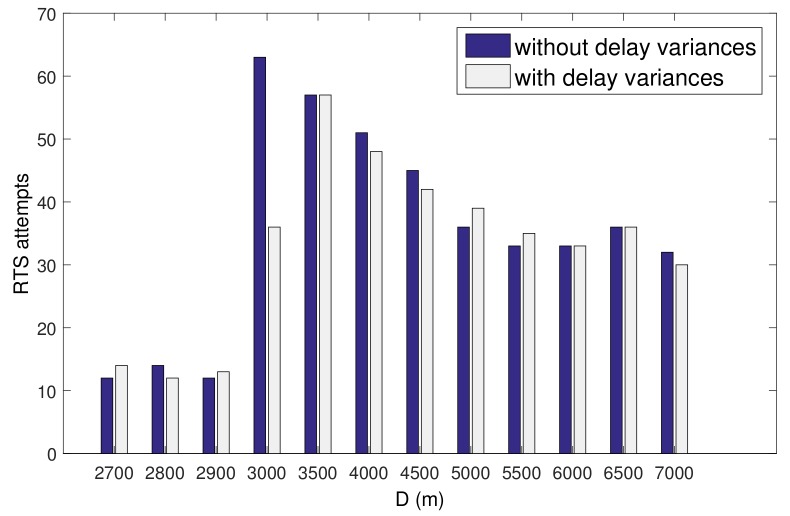
RTS attempts in different settings of the slot length.

The result of the RTS attempts in Figure 7 can explain the above throughput degradation. In the case of d<2 s, only a small amount of RTSs are sent, but no throughput is obtained. The slot length setting in this case results in the failures of RTS/CTS handshakes since no CTS response can be received within an RTT. The RTS/CTS handshake failures lead to the back-off for RTS retransmissions. The RTS attempts reach the maximum values at d=2 s. RTS attempts decrease in the case of d>2 s because the long slot length reduces the transmission opportunities and lead to throughput degradation.

### 5.3. Performance Comparisons

In this subsection, we compare our proposed Slotted-FAMA with adaptive slot length and the original Slotted-FAMA. We also plot the theoretical results in the same figure to verify our analytical model. We place seven nodes in a line with an interval of 500 m. The receiver node locates at the beginning of the line. The first transmitter locates at a distance of 500 m from the receiver, the second transmitter is located 1000 m from the receiver, and so on. Thus, the largest internodal distance in the network is 3000 m, and $d_{max}$ in Equation (1) for Slotted-FAMA should be set to 2 s. The small-scale delay variation is selected uniformly from −300 ms to 300 ms.

The small-scale delay variation is selected uniformly from [−300 ms, 300 ms] due to the low underwater acoustic propagation speed; a small drift of 100 m has led to a delay variance of ~67 ms at the speed of 1500 ms. In addition to the drift of nodes, the change of the propagation speed also leads to the dynamic of the propagation delay. The underwater acoustic propagation speed is changing in a range of [1450, 1540] due to the differences in salinity, temperature, depth, etc. There may be ~200 ms delay variance for 5 km from 1450 m/s to 1540 m/s.

**Figure 8:**
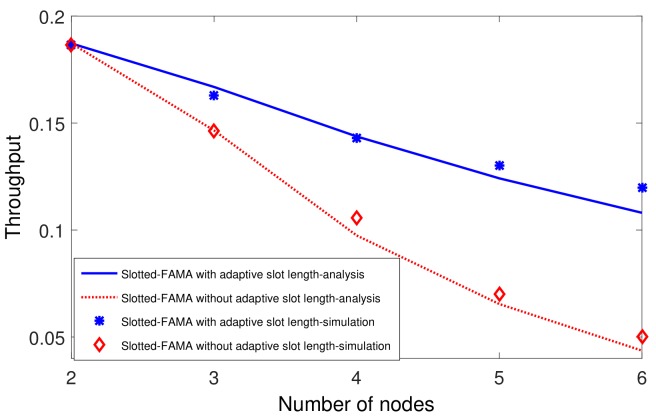
Throughput comparison for Slotted-FAMA with and without adapting the slot length in the network with different nodes.

We first compare the throughput in the network with different numbers of sending nodes. The average packet arrival rate is set as 0.3 packet/s. As shown in Figure 8, the throughput is deceased as the number of nodes in the network increases. That is because more nodes will introduce severe collisions for transmissions. Our proposed slot length adaptation improves the throughput significantly comparing to the fixed $d_{max}$ in the original Slotted-FAMA, especially in a network with a large number of nodes. Specially, our proposed adaptive slot length almost doubles the throughput of Slotted-FAMA, from 0.06 to 0.12, when the number of nodes is 6.

**Figure 9:**
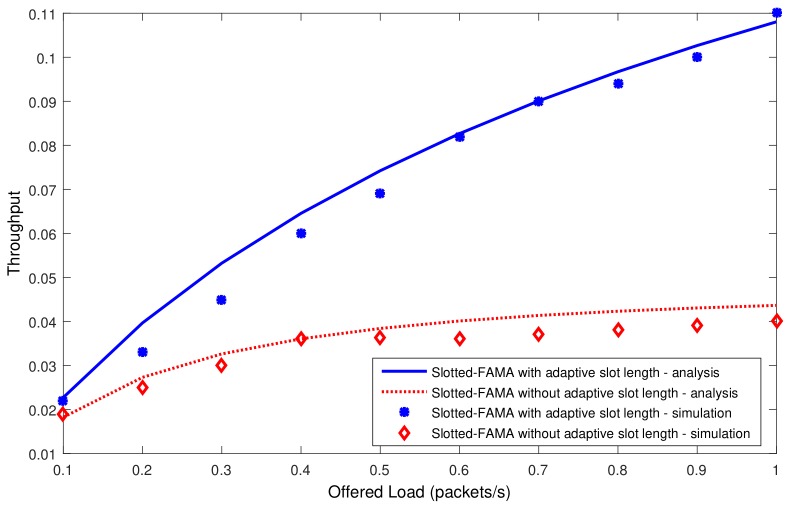
Throughput comparison for Slotted-FAMA with and without adapting the slot length in the network with different loads.

We next compare the throughput in the network with different loads. We vary the packet arrival rates from 0.1 packet/second to 1 packet/second. Figure 9 shows that the throughput improvement of Slotted-FAMA by our proposed adaptive slot length becomes increasing significant as the offered load increases. The throughput is increased from 0.04 to 0.11 when the offered load reaches to 1 packet/s.

## 6. Conclusions

This paper identified the existence of multiscale delay variations in underwater acoustic channels, including both large-scale and small-scale delay variations, and studied the impact of delay variation on the handshake based MAC protocols. The simulation on the slot length of Slotted-FAMA showed that our proposed slot length adaptation improves the efficiency of the channel reservation for the handshake- and slotting-based media access control. We have also established an absorbing Markov chain model to derive the closed-form expression for throughput of MAC. Both our analysis and simulation results reveal that the delay variation affects the setting of MAC protocols and decreases their throughput. Our proposed slot length adaptation increases significantly the throughput of Slotted-FAMA under multiscale delay variations.

The work in progress is to design an approach to estimate the propagation delay to further improve the MAC performance.

## Figures and Tables

**Table 1:** System notation lists.

Notation	Description
λ	Data generation rate
$K_{c}$	The probability of correctly receiving the control frame
$K_{d}$	The probability of correctly receiving the data frame
$T_{c}$	The duration of a control frame
$T_{d}$	The duration of a data frame
$T_{slot}$	A slot length

**Table 2:** State description.

States	Description	Stay Time
state 1	waiting packet arrivals and contending with back-off	$T_{cont}$
state 2	overhearing an xRTS ${}^{1}$	$T_{revXRTS}$
state 3	overhearing an xCTS after the corresponding xRTS	$T_{revXCTS}$
state 4	overhearing an xCTS	$T_{revXCTS}$
state 5	overhearing an xDATA	$T_{revXDATA}$
state 6	overhearing an xNACK	$T_{revXNACK}$
state 7	waiting for CTS	$T_{sndRTS}$
state 8	sending data packet	$T_{revCTS}$
state 9	receiving ACK	absorbing state

**Table 3:** State transition probabilities.

P(m,n)	Description
a	The probability of sending RTS packet
b	The probability of not receiving CTS packet
d	The probability of being idle
e	The probability of receiving CTS packet
f	The probability of overhearing xNACK packet
g	The probability of overhearing xDATA packet
h	The probability of overhearing xCTS packet
k	The probability of overhearing xRTS packet
p	The probability of overhearing xCTS packet after overhearing the corresponding xRTS packet
s	The probability of receiving ACK packet
